# Supplementary material for: Explainable AI toward understanding the performance of the top three TADPOLE Challenge methods in the forecast of Alzheimer’s disease diagnosis
Source: PLoS One. 2022 May 6;17(5):e0264695. doi: 10.1371/journal.pone.0264695 (PMC9075665; doi:10.1371/journal.pone.0264695)
Supplement: S1 File — Referred in the manuscript as supplementary material. (PDF) [file pone.0264695.s001.pdf]

Supplementary material of Explainable AI toward understanding the performance of the top-three TADPOLE challenge methods in the forecast of Alzheimer's disease diagnosis

Monica Hernandez [1], Ubaldo Ramon-Julvez [1], Francisco Ferraz [1] with the ADNI Consortium¶

1 Aragon Institute on Engineering Research, University of Zaragoza, Spain

¶Data used in preparation of this article were obtained from the Alzheimer's Disease Neuroimaging Initiative (ADNI) database (adni.loni.usc.edu). As such, the investigators within the ADNI contributed to the design and implementation of ADNI and/or provided data but did not participate in the analysis or writing of this report. A complete listing of ADNI investigators can be found at: http://adni.loni.usc.edu/wp-content/uploads/how_to_apply/ADNI_Acknowledgement_List.pdf.
* mhg@unizar.es

Abstract

This document accompanies our manuscript titled *Explainable AI toward understanding the performance of the top-three TADPOLE challenge methods in the forecast of Alzheimer's disease diagnosis* with some results complementing the study.

1 Understanding SHAP explanations on Titanic dataset

Fig 1 shows typical examples of SHAP values representation for a given problem (survival classification in Titanic dataset). The horizontal bar plot shows the mean absolute SHAP values of the most relevant features for survival classification. The plot shows that gender is the most relevant feature for the correct classification of survivals, followed by the title code of the person, and the class. These results are coherent with our knowledge of Titanic's tragedy. Therefore, the underlying machine learning method is able to generate a model aware of the outcome of survival due to the human decisions made during the disaster.

The violin plot in Fig 1 represents the impact of the feature values on the probability computed for the survival class by the model. The color code indicates the feature values for the different test samples, and it is useful to relate whether the high or low probabilities computed by the model are favored by given feature values. The color bar ranges from blue tones for low values to red tones for high values. Thus, the model favors high values of the probability of survival for high values of gender (i.e. woman). If we look at the class feature, we can see that the model favors high values of the probability of survival for low values of class (i.e. 1st class). In our study, we will use similar reasoning to establish the relevance of the features in the identification of the different classes (AD, MCI, or CN).

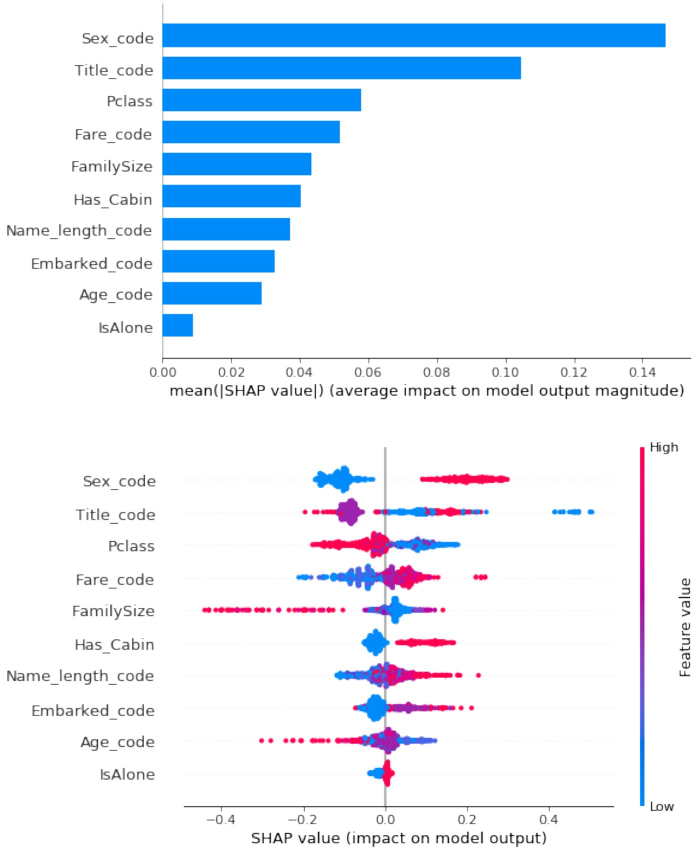

S1 Fig 1. SHAP explanations on Titanic dataset. Top, horizontal bar plot with feature importance. Down, violin plots representing the impact of the feature values in the probability of survival. Figures reproduced from `https://www.kaggle.com/meliao/shap-on-titanic-why-is-rose-alive-but-jack-not`.

2 Robustnes to sample size selection

Tables 1, 2, and 3 show the mean and standard deviation of the mAUC and BCA obtained by the methods in D2 and D4. In general, the metrics show a low standard deviation in both D2 and D4 with variations in the third decimal position in the majority of cases. Therefore, all the models obtain consistent results for the same configuration of sample and feature selection. The models trained with a 25% sample of the underrepresented class obtain metrics slightly under but close to the models trained with the whole underrepresented class. Therefore, the performance obtained by the methods can be considered robust to these changes on data sampling. The interpretability study proceeds now for the 100% sample sets.

3 Removing diagnosis, image, and cognitive features

Fig 2, 3, and 4 show the impact of each feature on the different model outputs for each class in the shape of violin plots. As happened with Figure 6 in the main manuscript, the different models are able to establish a relationship between feature values and diagnosis probability consistent with clinical knowledge.

S1 Table 1. XGB performance results.

	Test set (D2)		Evaluation set (D4)	
	mAUC	BCA	mAUC	BCA
XGB, 100%	0.998 (± 0.000)	0.982 (± 0.006)	0.909 (± 0.004)	0.804 (± 0.003)
XGB-D, 100%	0.945 (± 0.001)	0.863 (± 0.009)	0.897 (± 0.004)	0.788 (± 0.010)
XGB-D$_{ADNI}$, 100%	0.920 (± 0.005)	0.833 (± 0.005)	0.881 (± 0.005)	0.795 (± 0.008)
XGB-D-C, 100%	0.791 (± 0.008)	0.680 (± 0.013)	0.732 (± 0.011)	0.646 (± 0.016)
XGB-D-I, 100%	0.942 (± 0.004)	0.873 (± 0.003)	0.909 (± 0.006)	0.820 (± 0.009)
XGB, 50%	0.999 (± 0.002)	0.995 (± 0.004)	0.901 (± 0.006)	0.797 (± 0.006)
XGB-D, 50%	0.947 (± 0.004)	0.861 (± 0.013)	0.898 (± 0.005)	0.794 (± 0.007)
XGB-D$_{ADNI}$, 50%	0.918 (± 0.003)	0.835 (± 0.008)	0.878 (± 0.005)	0.797 (± 0.010)
XGB-D-C, 50%	0.789 (± 0.013)	0.688 (± 0.009)	0.732 (± 0.015)	0.649 (± 0.013)
XGB-D-I, 50%	0.940 (± 0.006)	0.872 (± 0.008)	0.909 (± 0.004)	0.824 (± 0.013)
XGB, 25%	0.999 (± 0.000)	0.996 (± 0.003)	0.899 (± 0.011)	0.799 (± 0.006)
XGB-D, 25%	0.945 (± 0.005)	0.860 (± 0.014)	0.895 (± 0.008)	0.788 (± 0.016)
XGB-D$_{ADNI}$, 25%	0.913 (± 0.005)	0.821 (± 0.017)	0.871 (± 0.008)	0.781 (± 0.013)
XGB-D-C, 25%	0.773 (± 0.014)	0.665 (± 0.013)	0.713 (± 0.012)	0.624 (± 0.016)
XGB-D-I, 25%	0.938 (± 0.004)	0.870 (± 0.013)	0.903 (± 0.005)	0.807 (± 0.010)

Models built with different feature sets: whole, without clinical diagnosis (-D), without clinical diagnosis and ADNI features for diagnosis (-D-D$_{ADNI}$), without clinical diagnosis and cognitive features (-D-C), and without clinical diagnosis and image features (-D-I). The training sets are balanced and use the 100% of the underrepresented class (AD). For each method configuration, it is shown the mean and standard deviation of the mAUC and BCA obtained over ten experiments.

S1 Table 2. RF performance results.

	Test set (D2)		Evaluation set (D4)	
	mAUC	BCA	mAUC	BCA
RF, 100%	0.976 (± 0.003)	0.908 (± 0.008)	0.894 (± 0.004)	0.804 (± 0.008)
RF-D, 100%	0.933 (± 0.003)	0.832 (± 0.008)	0.880 (± 0.005)	0.776 (± 0.012)
RF-D$_{ADNI}$, 100%	0.904 (± 0.005)	0.795 (± 0.010)	0.864 (± 0.006)	0.759 (± 0.008)
RF-D-C, 100%	0.774 (± 0.006)	0.671 (± 0.013)	0.721 (± 0.010)	0.625 (± 0.016)
RF-D-I, 100%	0.945 (± 0.003)	0.878 (± 0.011)	0.900 (± 0.004)	0.803 (± 0.008)
RF, 50%	0.975 (± 0.003)	0.906 (± 0.016)	0.892 (± 0.004)	0.798 (± 0.009)
RF-D, 50%	0.930 (± 0.004)	0.835 (± 0.010)	0.882 (± 0.003)	0.783 (± 0.013)
RF-D$_{ADNI}$, 50%	0.898 (± 0.005)	0.791 (± 0.013)	0.862 (± 0.010)	0.757 (± 0.011)
RF-D-C, 50%	0.775 (± 0.011)	0.668 (± 0.002)	0.722 (± 0.011)	0.622 (± 0.020)
RF-D-I, 50%	0.945 (± 0.002)	0.882 (± 0.012)	0.903 (± 0.006)	0.812 (± 0.009)
RF, 25%	0.965 (± 0.005)	0.887 (± 0.013)	0.892 (± 0.005)	0.904 (± 0.009)
RF-D, 25%	0.924 (± 0.004)	0.823 (± 0.006)	0.892 (± 0.008)	0.783 (± 0.014)
RF-D$_{ADNI}$, 25%	0.891 (± 0.006)	0.781 (± 0.013)	0.857 (± 0.007)	0.748 (± 0.013)
RF-D-C, 25%	0.772 (± 0.011)	0.660 (± 0.023)	0.720 (± 0.010)	0.621 (± 0.018)
RF-D-I, 25%	0.946 (± 0.003)	0.882 (± 0.005)	0.904 (± 0.005)	0.813 (± 0.010)

Same legend than Table 1. The training sets are balanced and use the 100%, 50%, and 25% of the underrepresented class (AD). For each method configuration it is shown the mean and standard deviation of the mAUC and BCA obtained over ten experiments.

S1 Table 3. SVM performance results.

	Test set (D2)		Evaluation set (D4)	
	mAUC	BCA	mAUC	BCA
SVM, 100%	0.925 (± 0.002)	0.815 (± 0.006)	0.845 (± 0.003)	0.757 (± 0.003)
SVM-D, 100%	0.909 (± 0.002)	0.785 (± 0.007)	0.839 (± 0.003)	0.739 (± 0.005)
SVM-D_{ADNI}, 100%	0.893 (± 0.002)	0.784 (± 0.008)	0.830 (± 0.003)	0.732 (± 0.007)
SVM-D-C, 100%	0.773 (± 0.006)	0.656 (± 0.011)	0.721 (± 0.006)	0.619 (± 0.011)
SVM-D-I, 100%	0.917 (± 0.002)	0.816 (± 0.009)	0.877 (± 0.002)	0.777 (± 0.093)
SVM, 50%	0.911 (± 0.004)	0.796 (± 0.012)	0.840 (± 0.006)	0.742 (± 0.012)
SVM-D, 50%	0.891 (± 0.004)	0.770 (± 0.007)	0.829 (± 0.005)	0.722 (± 0.006)
SVM-D_{ADNI}, 50%	0.884 (± 0.003)	0.771 (± 0.009)	0.824 (± 0.003)	0.724 (± 0.008)
SVM-D-C, 50%	0.771 (± 0.008)	0.665 (± 0.009)	0.717 (± 0.008)	0.620 (± 0.006)
SVM-D-I, 50%	0.914 (± 0.002)	0.807 (± 0.007)	0.874 (± 0.005)	0.760 (± 0.011)
SVM, 25%	0.897 (± 0.009)	0.778 (± 0.006)	0.827 (± 0.006)	0.726 (± 0.007)
SVM-D, 25%	0.880 (± 0.004)	0.767 (± 0.009)	0.819 (± 0.006)	0.717 (± 0.008)
SVM-D_{ADNI}, 25%	0.870 (± 0.010)	0.759 (± 0.014)	0.808 (± 0.011)	0.714 (± 0.015)
SVM-D-C, 25%	0.763 (± 0.010)	0.662 (± 0.015)	0.714 (± 0.010)	0.625 (± 0.017)
SVM-D-I, 25%	0.914 (± 0.004)	0.819 (± 0.015)	0.878 (± 0.006)	0.764 (± 0.011)

Same legend than Table 1. The training sets are balanced and use the 100%, 50%, and 25% of the underrepresented class (AD). For each method configuration it is shown the mean and standard deviation of the mAUC and BCA obtained over ten experiments.

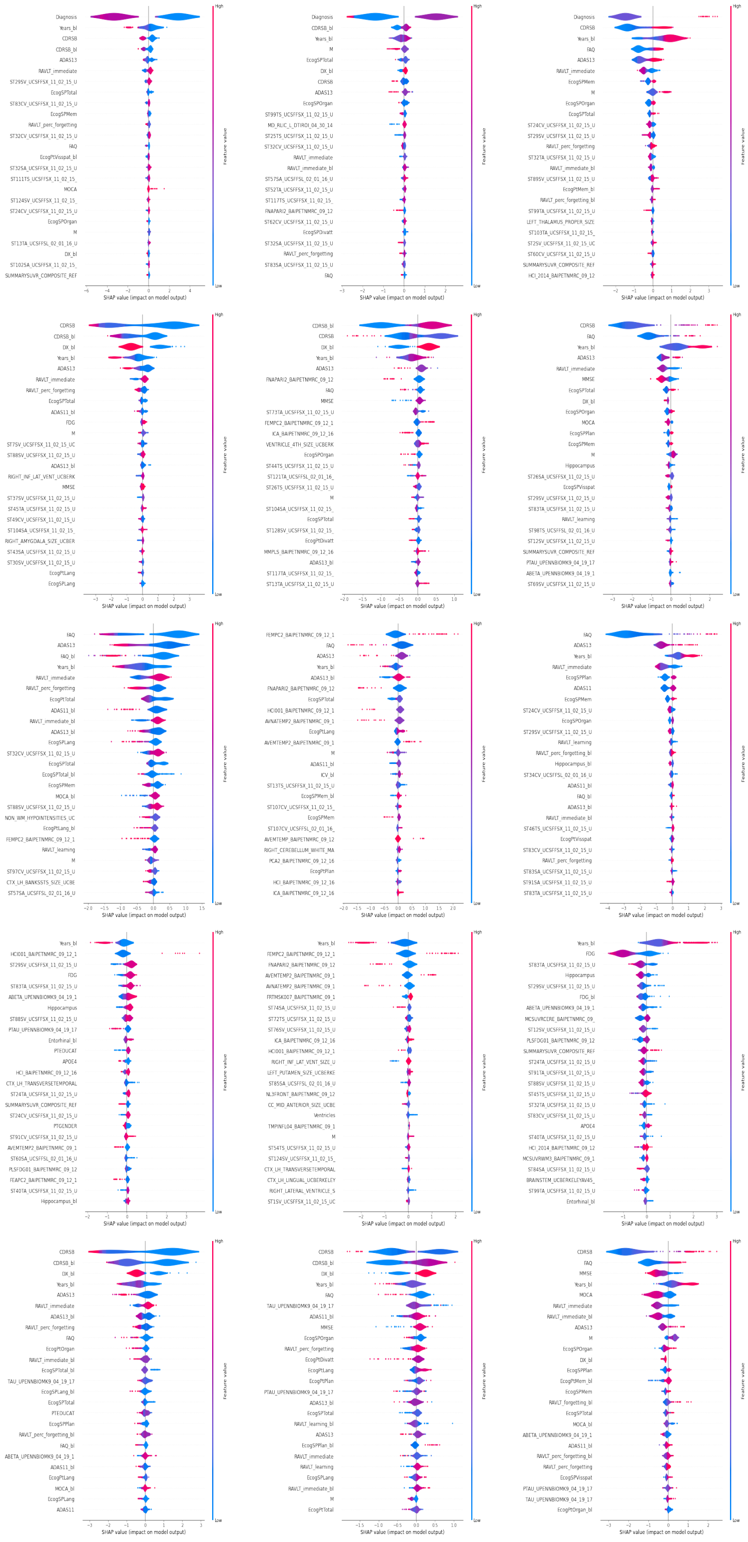

S1 Fig 2. XGB explainability. In the shape of violin plots, SHAP value impact on the estimation of the probabilities for each class performed by the XGB models. Each row shows the results for the considered feature sets (whole, -D, -D$_{ADNI}$, -D-C, and -D-I). Each column shows the results for each class. Long feature names have been trimmed for improving legibility.

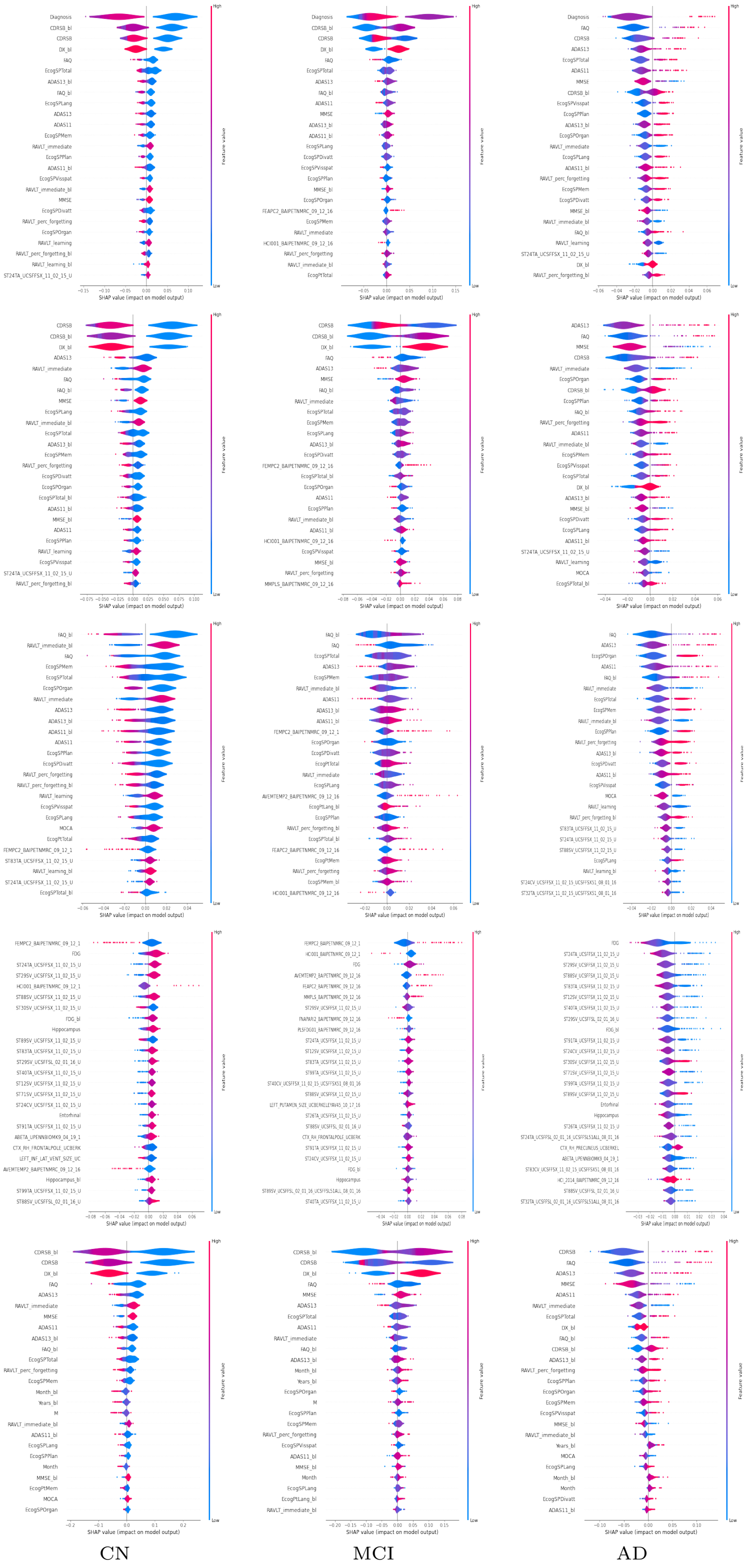

S1 Fig 3. RF explainability. In the shape of violin plots, SHAP value impact over the estimation of the probabilities for each class performed by the RF models. Each row shows the results for the considered feature sets (whole, -D, -D$_{ADNI}$, -D-C, and -D-I). Each column shows the results for each class. Long feature names have been trimmed for improving legibility.

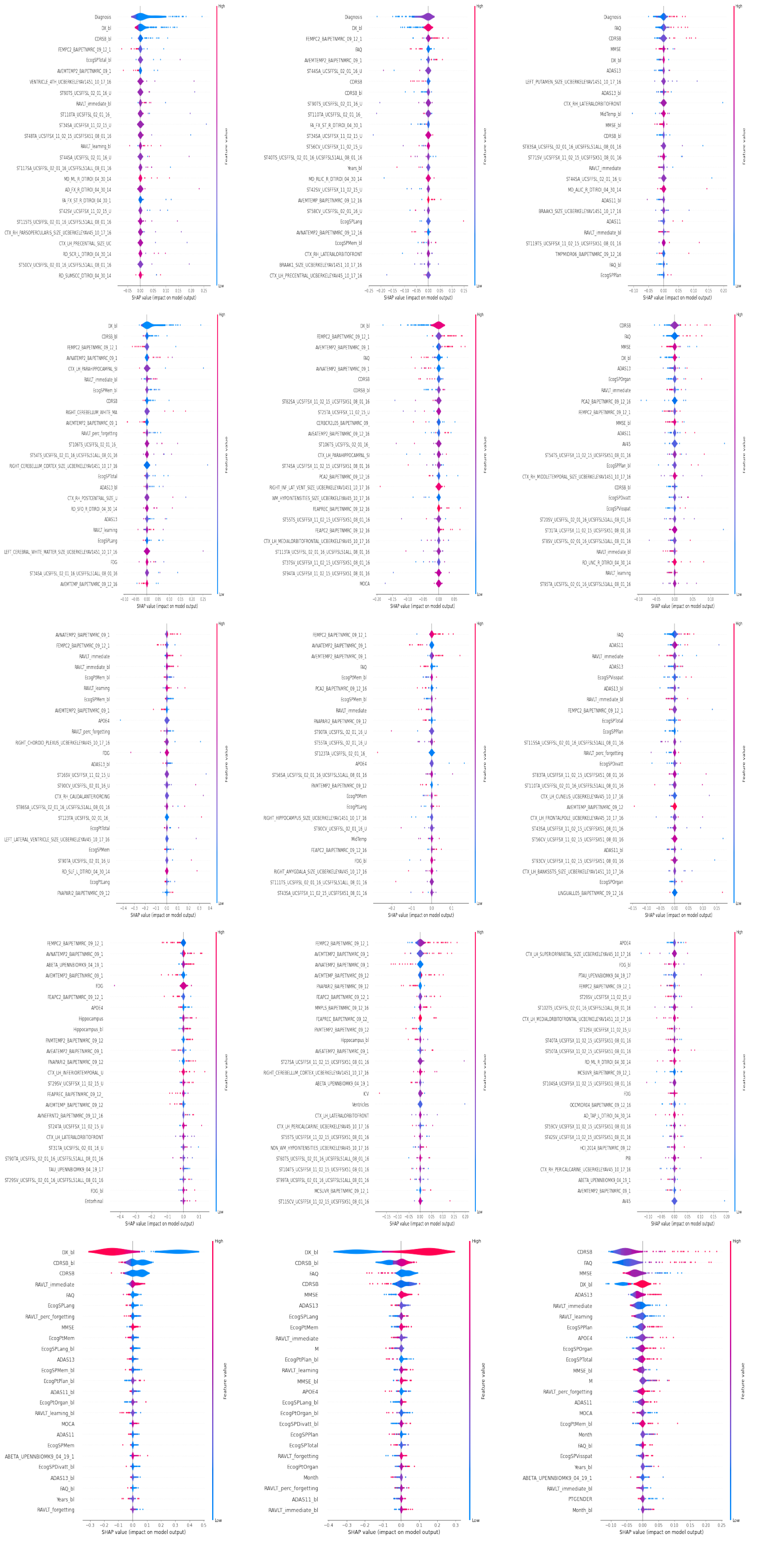

CN MCI AD

S1 Fig 4. SVM explainability. In the shape of violin plots, SHAP value impact over the estimation of the probabilities for each class performed by the RF models. Each row shows the results for the considered feature sets (whole, -D, -D$_{ADNI}$, -D-C, and -D-I). Each column shows the results for each class. Long feature names have been trimmed for improving legibility.
