# Supplementary material for: Explainable AI toward understanding the performance of the top three TADPOLE Challenge methods in the forecast of Alzheimer’s disease diagnosis
Source: PLoS One. 2022 May 6;17(5):e0264695. doi: 10.1371/journal.pone.0264695 (PMC9075665; doi:10.1371/journal.pone.0264695)
Supplement: Attachment — Submitted filename: PLOSOneR1Rebuttal.pdf [file pone.0264695.s005.pdf]

Response to the reviewers

We want to thank the reviewers for their work in the review process. We believe that their thorough comments helped us to improve the quality of the manuscript. We have revised the paper in accordance with their suggestions to the best of our ability. We tried to answer all the concerns raised by the reviewers. We made a particular effort in improving the English style of the manuscript by using Editage services. We also made an effort on improving the readability of the figures. We hope that the revised version of the manuscript will now meet the standards required for publication.

We would like to thank Reviewer 1 pointer to the recent state-of-the-art in explainable and actionable AI. In addition, we believe that the color-coding idea given by Reviewer 3 helped improve the readability of the figures. We would like to thank Reviewer 4 for his/her suggestion on our study limitation.

In the following pages, we include a point-by-point summary of our responses to the comments. Reviewer's points are presented in italics, while our responses are presented in normal text after "Response:". In the modified version of the manuscript, the corrected text is colored in blue.

Reviewer #1:

Summary

In this paper, the authors report on the TADPOLE (Alzheimer's Disease Prediction Of Longitudinal Evolution) project. The aim of this project is to identify the most predictive data, features and methods for the progression of patients at risk of developing Alzheimer's disease.

The challenge was successful in uncovering tree-based ensemble methods such as gradient boosting or random forests as the best methods for predicting the clinical state of Alzheimer's disease. However, the outcome of the competition was limited to which combination of data processing and methods had the best accuracy, making the contribution of methods to accuracy difficult to isolate. The quantification of feature importance was global of all methods used by the contest participants. In addition, Tadpole provided general answers aimed at improving performance, neglecting important aspects such as interpretability.

In this paper, the authors describe the models of the three best Tadpole methods in a common framework with the specific aim of providing a fair comparison. Furthermore, this paper also deals with explanations, in particular plausible explanations, why the methods have achieved this accuracy. For this purpose, the authors used well-known methods such as SHAP.

In this paper, the authors show that the two top-tadpole challenge methods are able to correctly determine those features most important for disease prognosis. This is good work, because it also reinforces confidence in such systems.

This reviewer finds this work important, relevant and interesting and would recommend acceptance and provides some recommendations for improvement below:

Main Points

1a) Check the overall language, maybe let an English native speaker help to get a better flow, the content is good and can benefit from a nicer flow.

Response: We hired Editage services to improve the overall language (https://www.editage.com/). Editage's editor provided a complete revision of the paper and we translated all his suggestions to the manuscript. We expect that our work can now be better followed up without English mistakes. Since the editing was exhaustive we did not color these changes.

1b) Check the wording e.g. section 1, page 2 multi-modal -> both high-dimensional (many data points) and multi-modal (from different sources)

Response: We included the reviewer's suggestion in the manuscript. Thank you very much for improving the meaning of the sentence.

1.2) Table 1 is exceeding margins

Response: The LaTeX template from PLOS One produces the page layout shown in Figure 1.2.1. The tables are apparently allowed to exceed margins in the LaTeX documentclass *article*. The page layout of our manuscript including Table 1 is shown in Figure 1.2.2.

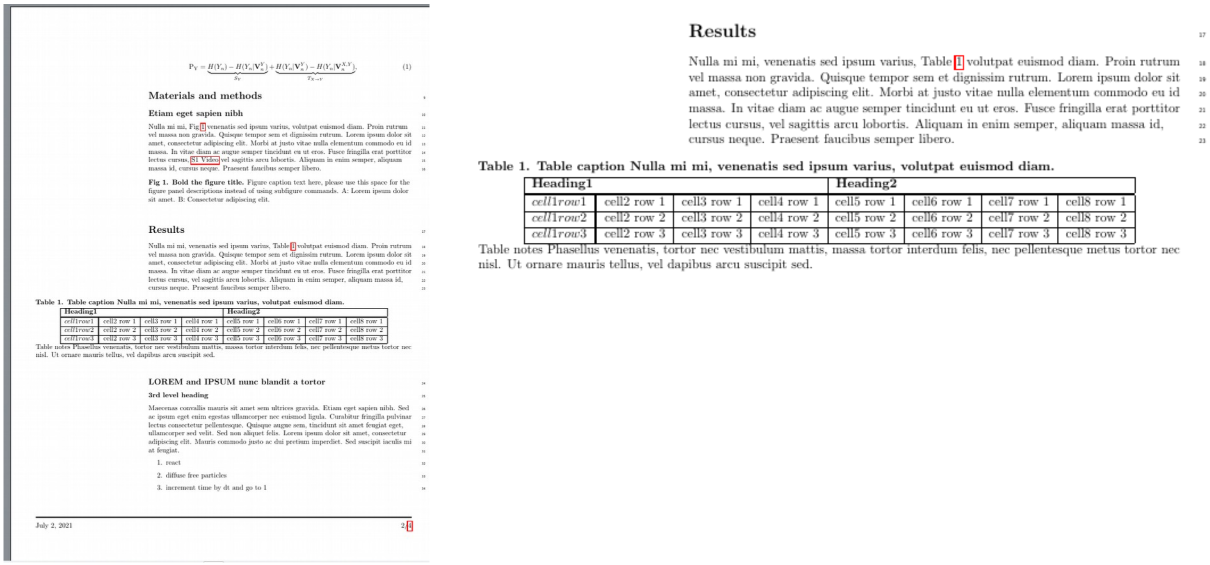

Results

Nulla mi mi, venenatis sed ipsum varius, Table 1 volutpat euismod diam. Proin rutrum vel massa non gravida. Quisque tempor sem et dignissim rutrum. Lorem ipsum dolor sit amet, consectetur adipiscing elit. Morbi at justo vitae nulla elementum commodo eu id massa. In vitae diam ac augue semper tincidunt eu ut eros. Fusce fringilla erat porttitor lectus cursus, vel sagittis arcu lobortis. Aliquam in enim semper, aliquam massa id, cursus neque. Praesent faucibus semper libero.

Table 1. Table caption Nulla mi mi, venenatis sed ipsum varius, volutpat euismod diam.

Heading1				Heading2			
cell1row1	cell2 row 1	cell3 row 1	cell4 row 1	cell5 row 1	cell6 row 1	cell7 row 1	cell8 row 1
cell1row2	cell2 row 2	cell3 row 2	cell4 row 2	cell5 row 2	cell6 row 2	cell7 row 2	cell8 row 2
cell1row3	cell2 row 3	cell3 row 3	cell4 row 3	cell5 row 3	cell6 row 3	cell7 row 3	cell8 row 3

Table notes Phasellus venenatis, tortor nec vestibulum mattis, massa tortor interdum felis, nec pellentesque metus tortor nec nisl. Ut ornare mauris tellus, vel dapibus arcu suscipit sed.

Figure 1.2.1. Layout of PLOS One LaTeX template including a table.

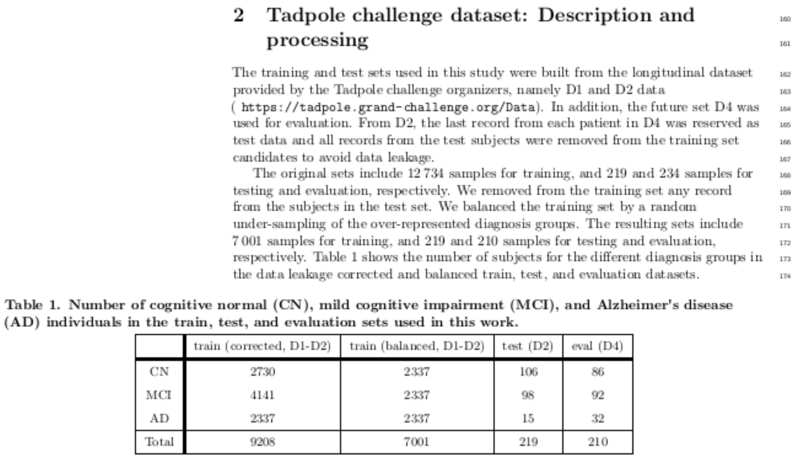

2 Tadpole challenge dataset: Description and processing

The training and test sets used in this study were built from the longitudinal dataset provided by the Tadpole challenge organizers, namely D1 and D2 data (https://tadpole.grand-challenge.org/Data). In addition, the future set D4 was used for evaluation. From D2, the last record from each patient in D4 was reserved as test data and all records from the test subjects were removed from the training set candidates to avoid data leakage.

The original sets include 12 734 samples for training, and 219 and 234 samples for testing and evaluation, respectively. We removed from the training set any record from the subjects in the test set. We balanced the training set by a random under-sampling of the over-represented diagnosis groups. The resulting sets include 7 001 samples for training, and 219 and 210 samples for testing and evaluation, respectively. Table 1 shows the number of subjects for the different diagnosis groups in the data leakage corrected and balanced train, test, and evaluation datasets.

Table 1. Number of cognitive normal (CN), mild cognitive impairment (MCI), and Alzheimer's disease (AD) individuals in the train, test, and evaluation sets used in this work.

	train (corrected, D1-D2)	train (balanced, D1-D2)	test (D2)	eval (D4)
CN	2730	2337	106	86
MCI	4141	2337	98	92
AD	2337	2337	15	32
Total	9208	7001	219	210

Figure 1.2.2. Layout of our manuscript including Table 1.

1.3) Section 3.2, page 7 To make this paper up-to-date, authors could also mention a very recent work on actionable xAI with RF, see: https://arxiv.org/abs/2108.11674

Response: We included a sentence regarding the missing link between explainable AI (the methods cited in the paper) and actionable explainable AI (the citation suggested by the reviewer) and a comment on alternatives for dealing with multi-modal information provided in the citation suggested by the reviewer. We consider that the changes should be performed in the Introduction instead of Section 3.2.

1.4) Section 4, page 9, when introducing xAI the authors should also introduce the importance of the quality of explanations particularly in a multi-modal setting, there is a very recent related work which can be helpful here, see: https://doi.org/10.1016/j.inffus.2021.01.008

Response: We would like to thank the reviewer for this interesting piece of work linking explainability with trustable and actionable systems for aiding decision-making in clinical applications. We included in Section 4 a paragraph condensing the ideas provided in that citation. We also included some other citations to related works on causability.

1.5) Attention, Figure 1 is unreadable and exceeds margin

Response: We changed the subfigure organization from horizontal to vertical and reduced the size of the figures to respect margins.

1.6) Figures 2 to 4 are difficult to read - maybe enlarge?

Response: We have transposed the layout of Figures 2 to 4. This way we have been able to enlarge the figures by 10% of the textwidth.

1.7) All the further figures the same- the reviewer does not know how the solution could be here ? But take care that the reader is not confronted with difficult readability.

Response: We have changed the layout of Figure 5 from a horizontal to a vertical arrangement. This way we have been able to enlarge the figures by 10% of the textwidth. We could not further enlarge since we are limited by the textheight also.

For the remaining figures (Figure 6 to 10), we were not able to find a satisfactory solution. We first considered further trimming the labels. However, some substrings of important labels were removed hindering a correct identification of the label (e.g. different RAVLT scores). We also considered a layout arrangement change, as it worked in Figures 2 to 4. However, the textheight limitation forced the use of exactly the same figure size as originally. Finally, we considered the rearrangement and combination of different Figures. However, the textheight limitation also forced us to keep the original figure size.

For Figures 6 and 7 we could not find any better solution than reading the digital version of the manuscript with zooming. Labels can be perfectly read with a zoom of 300%. We hope this would be enough for the reader to be able to analyze the interesting information.

For Figures 8, 9, and 10 we have ordered the features with the same frequency by alphabetical order and we have included the frequency results in the csv files of the supplementary material:

XGB_Summary_Plot_Robustness.csv, RF_Summary_Plot_Robustness.csv, and SVM_Summary_Plot _Robustness.csv, respectively. Thus, the interested reader can directly analyze the information regarding the frequency of features in the top 25.

Finally, we used the Preflight Analysis and Conversion Engine (PACE) digital diagnostic tool, https://pacev2.apexcovantage.com/ provided by PLOSOne in order to ensure that the figures meet PLOS requirements.

Reviewer #2:

Unfortunately, explainable AI means a more general concept. XAI should explain why technical findings contribute to medical findings. This reviewer cannot see any discussion on the biological aspects of AD derived from methods but a mere technical discussion.

Response: We recall the definitions found in the article kindly pointed out by reviewer 1 *https://doi.org/10.1016/j.inffus.2021.01.008*

Explainability ≔ technically highlights decision relevant parts of machine representations and machine models i.e., parts which contributed to model accuracy in training, or to a specific prediction for a particular observation. Here the xAI community has already developed a variety of successful methods. Explainability does not refer to a human model.

Causality ≔ the relationship between cause and effect in the sense of Judea Pearl.

Causability ≔ the measurable extent to which an explanation – resulting from (A) – to a human expert achieves a specified level of causal understanding. This can be measured e.g. with the System Causability Scale. Causability refers to a human model.

According to the first definition, we believe that our use of explainability is correct. We focused on the importance given to the features by the different models and verified whether the features arising in the top list are consistent with clinical knowledge. The way of using XAI methods was technical, no human model was involved in the process. Of course, our contribution is a first step towards actionable explainable methods. With our approach, we can only question the reliability of SVM, since the model strongly changes the importance given to different features among different bootstraps. In addition, we find that random forests mostly rely on cognitive features and this may be a problem for models aiming at early diagnosis.

Since the 1818 features are related to the biological aspects of AD we could only provide a ranking of features according to their importance in model decisions. In a setting with features related and not related to the biological aspects of AD or unknown, our proposed methodology may be used to assess whether the best performing models are able to ignore non-important features or give credit to some unknown feature to the date. This information would result in an increase or decrease of trust in the systems and features.

The connection of the biological aspects of AD and our technical explanations would fit into causability and actionability, where, with the help of a clinical specialist, we would evaluate the quality of our explanations and establish whether the explanations of the model enable some causal understanding of the disease. This could be a huge step towards computer- aided diagnosis of AD but it is outside the scope of the present work.

Reviewer #3:

This is a well-designed and well-executed study of the three top models in the TADPOLE challenge to predict evolution in Alzheimer's disease with machine learning models.

Importantly, they have confirmed the ability of the top two models in the challenge to predict outcome. As part of the Explainable AI initiative they have identified the most important features that predict progression in Alzheimer disease.

This is important work since many proposed models for prognostication never get validated independently on the original dataset.

3.1. I would defer to the editor and authors, but it would seem that TADPOLE like SHAP is an acronym and should be capitalized throughout.

Response: We have replaced all appearances of Tadpole with TADPOLE in the manuscript. In addition, we have checked that SHAP is written in upper case all over the manuscript.

3.2. The word "antagonist" on line 35 (page 2) seems wrongly used. Seems like they mean "Clinical research to mitigate Alzheimer's disease has taken two different directions."

Response: We are sorry for our use of English in this sentence. The word antagonist makes perfect sense in the authors' mother tongue. We replaced the word antagonist with the author's suggestion.

3.3. The Glossary on pages 1-2 is very helpful. Perhaps a few more abbreviations from Figs 2-10 can be added.

Response: We followed the reviewer's suggestion and added to the glossary some of the most frequent features appearing in Figures 2 - 10. Thank you for the suggestion, we believe that the reader will find the list of terms very useful.

3.4. The interpretation of the Feature labels from Figs 2-10 is difficult--requiring readers to refer to the initial data dictionary online. I suggest this is an issue for the Editor and Authors to resolve.

Response: Actually, the interpretation of the feature labels can be found through the text of Section 5. We have focused on the labels related to the most relevant results. With the name of the corresponding label, we include the explanation of the kind of label and finer details. For example, in the paragraph

"The most relevant features include the diagnosis, the age of each patient at baseline (Years bl), and a number of measurements from cognitive tests (CDRSB, FAQ, ADAS13, RAVLT, CDRSB bl, DX bl, ADAS11, and ECog). Then, a number of measurements from anatomical structures obtained from FreeSurfer cross-sectional pipeline (UCSFFSX) arise, interleaved with ADNIMERGE image and other cognitive measurements. The anatomical structures include the amygdala (ST17SV), the hippocampus (ST29SV), the inferior and middle temporal regions (ST32TA, ST40TA), the entorhinal region (ST83CV), and the cuneus (ST23SA)."

we first mentioned labels from cognitive tests and then labels from MRI cross-sectional biomarkers. In addition, we detailed the structures associated with the biomarkers since they are relevant to the diagnosis of Alzheimer's Disease. The reader can complete the interpretation of the labels with the information given in the completed glossary or the information in the referred webpage https://github.com/swhustla/pycon2017-alzheimers-hack/blob/master/docs/data_dictionary.md.

3.5. The font on figures 2-10 are barely legible. Again I suggest that the Editor and Authors resolve this issue.

Response: We tried to solve this issue to the best of our ability. Please, see our response to Reviewer 1 comments on points 1.6 and 1.7.

3.6. Verifying is misspelled on line 878. I hope that authors will spell check the entire manuscript.

Response: We hired Editage services to improve the overall language (https://www.editage.com/). Editage's editor provided a complete revision of the paper and we translated all his suggestions to the

manuscript. We expect that our work can now be better followed up without English mistakes. Since the editing was exhaustive we did not color these changes.

3.7. Given the length of this manuscript, I am not sure the Titanic figure and discussion adds anything of value (Figure 1).

Response: In order to reduce the length of the manuscript, we moved the enlarged Titanic figure to the supplementary material. We believe that Figure 1 in the context of Titanic survivors is a good example for explaining how to interpret the information given in SHAP figures in a use case that is accessible even to non-specialists. We believe readers not used to SHAP may find it useful to understand first the interpretation of SHAP figures in a much simpler example and then move to understand the interpretation of SHAP figures in the context of Alzheimer's Disease.

3.8. Color coding some of the bars of Figures 3, 4, etc. by feature category (e.g. Cognitive, Imaging, Biomarker, etc) may highlight differences in feature selection more dramatically.

Response: Apart from the legibility improvement in response to point 1.7, we have used a color-coding for the bars of Figures 2, 3, 4, 8, 9, 10. We believe that this is a great idea that has improved the legibility of the plots and we thank the reviewer for the suggestion.

Reviewer #4:

The manuscript outlines a timely and important piece of research. The study is well contextualised in the coverage of the literature, and the need for explainable AI in medical imaging is well justified. The sections that follow provide a logical account of the work conducted, including useful discussions of the data, the preprocessing methods, the predictive models and the explainable AI methods used. Enough detail is provided to allow other researchers to replicate the process. The results are presented along with a thoughtful exploration of the importance of feature set selection and sample size. The subsequent analysis of interpretability is comprehensive, and supports the concluding remarks about the performance of the models, the most meaningful features, and the consistency between the models and clinical knowledge. In essence, this is a robust piece of work, but it is sometimes undermined by the presentation.

There are a number of flaws which should be addressed:

4.1). The authors should provide details on the analysis/justification of the validation strategy. A train/test/evaluation split is conducted – might k-fold cross-validation be considered in addition or as an alternative? Why was k-fold cross validation not used?

Response: In fact, k-fold cross-validation with 5 folds was used in the selection of the hyperparameters through the class RandomizedSearchCV https://scikit-learn.org/stable/modules/generated/sklearn.model_selection.RandomizedSearchCV.html. We have included this detail in the description of the TADPOLE challenge methods (Section 3).

Regarding our validation strategy, it was the one used in the TADPOLE challenge. The rationale behind this choice is to show that our models are able to obtain test and evaluation results similar to Frog, ThreeDays, and EMC-EB models while meeting the objective of isolating the contribution of pure machine-learning methods to the accuracy in diagnosis.

4.2). The authors should acknowledge the weakness in their validation. The test and evaluation data originate from the same study and distribution, and therefore the performance is unlikely to reflect the models' performance in a truly external dataset or clinical setting.

Response: We do agree with the reviewer that the evaluation and interpretability results of our models may not be preserved with different test and evaluation datasets. We believe that a very interesting direction would be to corroborate our findings with external clinical datasets. Towards this end, we are currently working with Dr. Antonio Lobo, from the Lozano Blesa University Hospital in our town Zaragoza in order to apply our methods to an in-house database of psychiatric records (ZARADEMP project, https://cheba.unsw.edu.au/consortia/cosmic/studies/zarademp-project).

We added a paragraph in the Discussion and Conclusions section of our manuscript acknowledging the limitation.

4.3). Intelligibility and standard of English. This is the area the manuscript has the most flaws: there are frequent grammar and spelling errors which obfuscate the meaning. These are really too numerous to list. The manuscript should be very carefully proofread by a native English speaker for errors to improve readability.

Response: We hired Editage services to improve the overall language (https://www.editage.com/). Editage's editor provided a complete revision of the paper and we translated all his suggestions to the manuscript. We expect that our work can now be better followed up without English mistakes. Since the editing was exhaustive we did not color these changes.

4.4). The plots are good, but there are many of them which means that they and their labels are small and condensed, making them difficult to inspect and compare. The authors should enlarge the plots. This may require rearrangement, being more selective about plot inclusion, or some sort of aggregation of plots.

Response: We tried to solve this issue to the best of our ability. Please, see our response to Reviewer 1 comments on points 1.6 and 1.7.
